# A study on the association between uncorrected visual acuity and 24-h activity behaviors among middle school students in Tianjin

**DOI:** 10.3389/fpubh.2026.1701765

**Published:** 2026-09-14

**Authors:** Yanan Zhao, Li Fang, Jinzhe Li, Liquan Cao, Sijie Tan

**Affiliations:** Tianjin Sport University, Tianjin Key Laboratory of Sports Health Integration and Health Promotion, Tianjin, China

**Keywords:** LASSO regression, middle school students, moderate-to-vigorous physical activity, myopia, sedentary behavior, sleep

## Abstract

**Objective:**

This study aims to investigate the independent and combined effects of 24-h activity behaviors—including sedentary behavior, light-intensity physical activity, moderate-to-vigorous physical activity (MVPA), and sleep—on myopia among middle school students in Tianjin. The findings are intended to inform strategies for myopia prevention and control, as well as the development of evidence-based physical activity prescriptions for children and adolescents.

**Methods:**

A stratified cluster sampling method is used to recruit 1,212 middle school students from five districts in Tianjin. Participants wore accelerometers (ActiGraph GT3X+) for seven consecutive days to objectively monitor their 24-h activity patterns. Ophthalmologic examinations are conducted to assess Uncorrected Visual Acuity. LASSO regression analysis is employed to explore the associations between activity behaviors and uncorrected visual acuity. Given the inherent interdependence and multicollinearity among time-based 24-h activity behaviors, LASSO regression was employed to enable robust variable selection and improve model stability compared with traditional regression approaches.

**Results:**

(1) Prevalence of Myopia: the overall myopia detection rate is 73.3%, with a higher prevalence among girls. Severe visual impairment accounted for the highest proportion (38.0%), and the prevalence increased significantly with grade level, reaching 39.2% in the third year of middle school. (2) 24-h Activity Behaviors: Daily sedentary time (572.08 ± 51.16 min) significantly exceeded recommended guidelines. Both MVPA (38.74 ± 15.36 min) and sleep duration (453.10 ± 47.18 min) are below the recommended levels. (3) Associations with Uncorrected Visual Acuity: LASSO regression indicated that MVPA (β = 0.162) and sleep duration (β = 0.131) are positively associated with uncorrected visual acuity, suggesting protective effects against myopia. In contrast, sedentary behavior (β = −0.106) and light-intensity physical activity (β = −0.078) are negatively associated with uncorrected visual acuity, implying an increased risk of myopia.

**Conclusion:**

Twenty-four-hour activity behaviors are significantly associated with uncorrected visual acuity in middle school students. Higher MVPA and longer sleep are associated with better visual acuity, whereas greater sedentary behavior and light-intensity physical activity are associated with poorer visual outcomes.

## 1 Introduction

Myopia, or nearsightedness, refers to a type of refractive error in which parallel light rays entering the eye are focused in front of the retina, resulting in blurred vision. It is often associated with genetic predisposition and poor visual habits. Of particular concern is that high myopia can lead to irreversible vision impairments such as retinal detachment and myopic maculopathy, which are becoming major causes of vision loss and blindness (1). Myopia is the most prevalent eye condition among children and adolescents and has increasingly become a global public health concern. According to the World Health Organization (WHO) and large-scale epidemiological studies, approximately 30% of the global population is currently affected by myopia, and this proportion is projected to increase to nearly 50% by 2050, with high myopia affecting about 10% (2). In China, the situation is particularly severe (3). According to a 2020 report by the National Health Commission of China (4), the overall prevalence of myopia among Chinese children and adolescents is 52.7%, with rates of 35.6% in primary school, 71.1% in middle school, and 80.5% in high school. These figures highlight not only the significant impact of myopia on individual physical and mental health but also the substantial economic burden on families.

Given the urgency of addressing myopia in youth, the Chinese Ministry of Education and other departments jointly issued the *Implementation Plan for Comprehensive Prevention and Control of Myopia in Children and Adolescents* in 2018 (5), which emphasized the need to establish and refine comprehensive prevention and control mechanisms. In recent years, a growing body of research has demonstrated that daily activity behaviors play a crucial role in the visual health of children and adolescents. These behaviors are typically captured under the concept of 24-h activity behavior, which includes sedentary behavior (SB), sleep (SLP), and physical activity (PA). PA can be further categorized into light-intensity physical activity (LPA) and moderate-to-vigorous physical activity (MVPA) (6). However, most previous studies have focused on the effect of a single behavior—such as SB or PA—on myopia (7), failing to consider the integrated and interdependent nature of 24-h movement behaviors. Moreover, most existing research has centered on the physical fitness implications of activity patterns, with relatively few studies directly addressing their relationship with myopia (8, 9).

Therefore, this study aims to investigate the independent and combined effects of the four components of 24-h activity behavior—SB, LPA, MVPA, and SLP—on myopia among children and adolescents. Importantly, this study emphasizes methodological advancement rather than the identification of novel directional associations alone. By objectively quantifying the full spectrum of 24-h activity behaviors using 7-day accelerometer-based monitoring and adopting an integrated 24-h movement behavior framework, this study aims to provide a more accurate and ecologically valid assessment of behavioral correlates of uncorrected visual acuity, in order to provide both theoretical insight and practical guidance for myopia prevention, health promotion, and the development of tailored physical activity prescriptions for youth.

## 2 Materials and methods

### 2.1 Participants

Tianjin was selected as a highly urbanized metropolitan city in northern China with a high prevalence of adolescent myopia. Standardized data collection was ensured through the local Student Physical Fitness Monitoring Program, and participants were recruited using stratified cluster sampling across urban and suburban districts. While specific to Tianjin, the sample characteristics support relevance to other comparable urban settings in China. The participants of this study are students from Grade 7 to Grade 9 (equivalent to the first through third years of junior high school) in Tianjin, China. The data collection is conducted between January and March 2024, Experiments (January 2024), data collection (February 2024), and data statistical analysis (March 2024) are all conducted in winter. During January's holiday, the experiment adopted a simulated non-vacation activity pattern (with consent from participants, guardians, and schools) to ensure homogeneous activity data, including aligning with non-vacation schedules and conducting structured physical activities. Relying on the physical fitness monitoring program for students in Tianjin, a stratified cluster sampling method is employed to select participants. The minimum required sample size is calculated using a standard sample size formula (10) (Formula 1 where n is the minimum required sample size; *u*α/2 denotes the critical value of the standard normal distribution at the chosen significance level (α = 0.05); π is the expected prevalence of myopia in the target population; and δ is the permissible absolute error of the estimate.), with an assumed myopia prevalence of 71.1% among junior high school students in China and a 10% non-response rate taken into account. Inclusion criteria: students in Grade 7 to Grade 9 who are able to complete the assessments. Exclusion criteria: students who are unable to undergo ophthalmologic examinations; those diagnosed with systemic or ocular conditions that affect eye development; students who failed to wear the accelerometer as instructed; and participants with incomplete or missing data.

$$\begin{array}{l} n=u2a/2\times \pi \left(1-\pi \right)/\delta 2 \end{array}$$ (1)

Sampling procedure: Five districts in Tianjin are randomly selected: heping District, Nankai District, Jinghai District, Xiqing District, and Hexi District. Two junior high schools are randomly selected from each district. Within each school, students from Grades 7–9 are randomly recruited. All participants and their guardians provided written informed consent. Students who voluntarily participated in the study completed the following steps: a *Student Physical Fitness and Health Questionnaire*; a 7-day monitoring of 24-h activity behaviors using an accelerometer; an ophthalmologic examination. A total of 1,256 students are initially recruited, among whom 44 are excluded due to missing or invalid accelerometer data. The study is approved by the Ethics Committee of Tianjin University of Sport (Ethics Approval No. TJUS2025-032).

### 2.2 Research methods

#### 2.2.1 Informed consent and questionnaire administration

The questionnaires used in this study are derived from the *National Student Physical Fitness and Health Survey Questionnaire* and the *Mental Health Inventory for Chinese Middle School Students*, both of which are employed in the Eighth National Survey on Students' Physical Fitness and Health. These instruments collected data on participants' demographic information, physical activity behaviors, general health status, and mental health. To evaluate the reliability and validity of the measurement scales within the questionnaire, IBM SPSS Statistics 27.0 is used:

Reliability Test: Cronbach's alpha coefficient is calculated to assess internal consistency. The Cronbach's α value for the measurement scales is 0.879, indicating *excellent reliability*. Validity Test: *Content Validity:* the questionnaire is designed based on rigorous theoretical foundations, revised and approved by subject-matter experts to ensure that the items accurately reflected the intended constructs. *Construct Validity:* the Kaiser-Meyer-Olkin (KMO) test and Bartlett's test of sphericity are conducted. The KMO value is 0.910 and Bartlett's test yielded *P* < 0.05, suggesting *excellent validity*.

#### 2.2.2 Assessment of academic stress

The academic stress component is assessed using relevant items from the *Mental Health Inventory for Chinese Middle School Students*. Reliability test: the Cronbach's α value for the academic stress subscale is 0.861, indicating high internal consistency. Validity test: *Content Validity:* the scale is theoretically grounded, expert-reviewed, and considered valid for assessing academic stress. *Construct validity:* the KMO value is 0.884 and Bartlett's test yielded *P* < 0.05, demonstrating good construct validity.

#### 2.2.3 Measurement of 24-H activity behaviors

The ActiGraph GT3X triaxial accelerometer is used to objectively measure 24-h activity behaviors of participants. Trained researchers instructed participants on how to wear the accelerometer and explained important precautions. Participants are required to wear the device continuously for seven consecutive days, covering five weekdays and two weekend days. The accelerometer is worn on the right hip, directly above the iliac crest, and is to be removed only during sleep, bathing, or similar activities. After the monitoring period, the devices are collected and data are processed using ActiLife 6.0 software. Physical activity (PA) and sedentary behavior (SB) durations are objectively assessed from the accelerometer data. Meanwhile, the study used the Physical Activity Questionnaire for Adolescents (PAQ-A) to conduct cross-validation of physical activity levels. A random subset of 20% of the participants (*n* = 242) is selected, and the consistency between accelerometer-measured physical activity duration and PAQ-A-reported results is analyzed using the intraclass correlation coefficient (ICC). The results showed strong consistency between the two measurements (ICC = 0.78, 95% confidence interval (95% CI): 0.72–0.83), supporting its use in subsequent analyses.

Sleep (SLP) duration is initially calculated by subtracting the sum of PA and SB from the total daily time (1,440 min per day). To verify the accuracy of this calculation, a cross-validation is conducted using the standardized sleep assessment questions from the National Student Physical Fitness and Health Survey Questionnaire [a tool with established reliability and validity in Chinese adolescent populations (relevant citation if available, e.g., national survey protocol or validated study)]. A random subset of 20% of participants (*n* = 242) is selected for cross-validation, and the consistency between accelerometer-derived sleep duration and questionnaire-reported sleep duration is analyzed using ICC. Results showed that the accelerometer-derived sleep duration is strongly consistent with questionnaire-reported sleep duration (ICC = 0.82, 95% CI: 0.76–0.87). Given this acceptable consistency, the initial accelerometer-derived sleep duration data are retained for subsequent analyses.

Key accelerometer parameters included (Table 1): sampling interval, non-wear time definition, valid wear days, minimum daily wear time, valid data criteria per day, and activity intensity cut-points for different physical activity levels.

**Table 1:** Accelerometer parameter settings.

Parameter	Setting
1. Device	ActiGraph GT3X+
2. Sampling interval	60 s
3. Non-wear time	Defined using the Choi algorithm
4. Valid wear time per day	≥600 min
5. Minimum valid days for inclusion	At least 4 days (including 3 weekdays and 1 weekend day)
Cut-off points for physical activity intensity levels	
SB/(counts/min)	< 100
LPA/(counts/min)	100–2,295
MVPA/(counts/min)	≥2,295

#### 2.2.4 Ophthalmologic examination

In accordance with the *Comprehensive Strategy for the Prevention and Control of Myopia in Children and Adolescents in Tianjin* (2019) (3), professional ophthalmologists and optometrists conducted all ophthalmologic assessments in a laboratory setting. The examination included both distance uncorrected visual acuity testing and refractive error assessment, and followed the steps outlined below: Pre-examination preparation: participants are asked whether they wear contact lenses or orthokeratology (ortho-k) lenses, and this information is recorded. those wearing contact lenses are instructed to remove them at least 30 min in advance to prevent interference with results, whereas those with ortho-k lenses are directly flagged for subsequent myopia classification. Uncorrected visual acuity (UCVA) test : distance visual acuity is measured using a standard electronic visual acuity chart placed 5 m in front of the examinee. Both uncorrected and corrected visual acuity are tested separately for the left and right eyes. The grades of UCVA: Normal Vision >5.0, Mild Visual Impairment 4.9, Moderate Visual Impairment 4.6–4.8, and Severe Visual Impairment ≤ 4.5. Refractive error measurement: refractive error is assessed using the Tianle RM-9600 automated refractometer under non-cycloplegic conditions. Each day, the device is calibrated using a standard model eye before testing, and cylindrical values are adjusted to negative form. Each eye is measured three times, and the average is recorded. If the difference in spherical equivalent (SE) between any two of the three readings is ≥0.05 diopters (*D*), additional measurements are taken until acceptable consistency is achieved. For those wearing contact lenses, lenses are removed at least 30 min prior to the refraction test. Myopia screening criteria: Based on the *Guidelines for Myopia Prevention and Control in China* (2024 edition) (11), a participant is classified as having screening-detected myopia if either eye had an uncorrected visual acuity of < 5.0 and a spherical equivalent (SE) of < −0.50 D. Individuals wearing orthokeratology lenses are automatically classified as myopic (12).

### 2.3 Quality control

This study is conducted at the Key Laboratory of Physical Education–Health Integration and Health Promotion in Tianjin. To minimize data errors during the testing process, all equipment is calibrated by the same technician prior to the start of data collection. Physical fitness assessments are carried out by professionally trained staff who had received standardized training to ensure consistency. Data entry is performed using a double-entry method by two independent personnel to ensure data accuracy and authenticity.

### 2.4 Statistical analysis

Data processing and statistical analyses are conducted using IBM SPSS Statistics version 27.0 and the glmnet package in R version 4.4.2. The specific methods are as follows: Descriptive and inferential statistics: for continuous variables that conformed to a normal distribution, results are expressed as *mean* ± *standard deviation* (SD). Independent samples *t*-tests are used to compare group differences. The normality of the uncorrected visual acuity results and 24-h activity behaviors is assessed using Quantile-Quantile (Q–Q) plots. Categorical variables are described using frequency counts or percentages. Comparisons among different grade levels, genders, and uncorrected visual acuity categories are conducted using chi-square (χ^2^) tests. Correlation analysis: Pearson correlation and multiple linear regression are used to explore the relationship between uncorrected visual acuity and 24-h activity behaviors. Correlation strength is interpreted as follows: |0–0.2|: very weak or no correlation |0.2–0.4|: weak correlation |0.4–0.6|: moderate correlation |0.6–0.8|: strong correlation |0.8–1.0|: very strong correlation. LASSO regression: prior to multivariate modeling, univariate analyses are performed to identify potential risk factors of impaired UCVA, variables with *P* < 0.05 in univariate analysis are included in the LASSO regression model; Least Absolute Shrinkage and Selection Operator (LASSO) regression (13) is employed to identify key 24-h activity behaviors that significantly impact uncorrected visual acuity among junior high school students.

$$\begin{array}{l} {min}_{\beta \left\{\frac{1}{2n}\overset{n}{\underset{i=1}{\sum }}{\left(y_{i}-x_{i}^{T}\beta \right)}^{2}+{\lambda ||\beta ||}_{1}\right\}} \end{array}$$ (2)

## 3 Results

### 3.1 Basic characteristics of participants

According to the results presented in Table 2, among all participants, the proportions of students reporting different levels of academic stress are as follows: mild (19.9%), moderate (44.1%), relatively high (25.5%), and severe (10.5%). As shown in Table 2, only 26.7% of the participants had normal uncorrected visual acuity, while 73.3% are identified as myopic. Among the myopic students, the largest proportion had severe visual impairment (38.0%), followed by moderate impairment (28.9%), and mild impairment (6.4%). Chi-square test results indicated that there are no statistically significant differences in the distribution of uncorrected visual acuity levels between male and female students (*P* > 0.05 for all comparisons). However, across the three grade levels, significant differences are observed in the number of students with normal vision and those with mild visual impairment (*P* < 0.05 for both comparisons) Table 3.

**Table 2:** UCVA information and comparison of the subjects (%).

Category	*n* (%)	Normal vision	Mild visual impairment	Moderate visual impairment	Severe visual impairment
Total	1,212	323 (26.7%)	77 (6.4%)	351 (28.9%)	461 (38.0%)
Gender					
Male	642 (53.0%)	184 (28.6%)	41 (6.4%)	188 (29.3%)	229 (35.7%)
Female	570 (47.0%)	139 (24.4%)	36 (6.3%)	163 (28.6%)	232 (40.7%)
*P*-value		0.093	0.96	0.792	0.072
Grade					
Grade 7	442 (36.5%)	138 (31.2%)	19 (4.3%)	117 (26.5%)	153 (37.0%)
Grade 8	357 (29.4%)	86 (24.1%)	24 (6.7%)	107 (30.0%)	168 (38.0%)
Grade 9	413 (34.1%)	99 (24.0%)	34 (8.2%)	127 (30.8%)	140 (39.2%)
*P*-Value		< 0.05	< 0.05	0.341	0.826

**Table 3:** Basic information and comparison of subjects (%).

Category	*n* (%)
Gender	
Male	642 (53.0%)
Female	570 (47.0%)
Residence	
Urban	742 (61.2%)
Rural	470 (38.8%)
Parental education level	
Below high school	368 (30.4%)
High school and above	844 (69.6%)
Parental myopia status	
Neither parent myopic	329 (27.1%)
One parent or both parents myopic	320 (26.4%)
Monthly household income (CNY)	
< 8,000	389 (32.1%)
≥8,000	823 (67.9%)
Academic stress level	
Mild	241 (19.9%)
Moderate	535 (44.1%)
Relatively high	309 (25.5%)
Severe	127 (10.5%)
Daily outdoor activity time	
< 30 min/day	734 (60.6%)
≥30 min/day	478 (39.4%)

### 3.2 24-h activity behaviors among middle school students by gender and grade level

As shown in Table 4, the average daily durations of 24-h activity behaviors are 38.74 ± 15.36 min for MVPA, 376.09 ± 17.16 min for LPA, 572.08 ± 51.16 min for SB, and 453.10 ± 47.18 min for SLP. Independent-samples *t*-tests showed significant gender differences in LPA and MVPA (*P* < 0.05), but not in SB or SLP (*P* > 0.05). One-way ANOVA indicated significant grade-level differences in all four activity behaviors (*P* < 0.01), suggesting that daily activity patterns vary notably across grades.

**Table 4:** Comparison of 24-h activity behavior of subjects with different genders and grades ($\bar{x}$ ± *s*).

Category	LPA (min)	MVPA (min)	SB (min)	SLP (min)
Total	376.09 ± 17.16	38.74 ± 15.36	572.08 ± 51.16	453.10 ± 47.18
Gender				
Male	375.14 ± 17.39	39.65 ± 15.60	571.48 ± 51.23	453.74 ± 47.18
Female	377.16 ± 16.85	37.71 ± 15.02	572.75 ± 51.12	452.38 ± 47.21
*P*-Value	< 0.05	< 0.05	0.665	0.616
Grade				
Grade 7	379.00 ± 16.59	39.89 ± 14.97	515.37 ± 17.67	505.74 ± 18.20
Grade 8	377.31 ± 20.82	39.38 ± 16.08	573.97 ± 19.26	449.34 ± 22.66
Grade 9	371.22 ± 10.54	36.61 ± 14.70	635.33 ± 18.37	396.85 ± 12.14
*P*-value	< 0.01	< 0.01	< 0.01	< 0.01

### 3.3 Association between uncorrected visual acuity and 24-h activity behaviors

Table 5 presents the associations between uncorrected visual acuity outcomes and 24-h activity behaviors across Grades 7 to 9. Overall, uncorrected visual acuity is negatively correlated with LPA and SB, and positively correlated with MVPA and SLP. Across all three grade levels, the correlation coefficients are all ≥ |0.7|, indicating a strong and statistically significant association between uncorrected visual acuity and the four components of 24-h activity behavior.

**Table 5:** Correlation between UCVA and 24 h activity behavior in different grades.

UCVA examination results	LPA	MVPA	SB	SLP
Grade 7 UCVA	−0.846	0.899	−0.904	0.909
Grade 8 UCVA	−0.830	0.888	−0.784	0.800
Grade 9 UCVA	−0.706	0.901	−0.918	0.911

Logistic regression analysis is performed to clarify the relationship between 24-h activity behaviors and the categorical outcome of myopia (myopic vs. non-myopic). As shown in Table 5, moderate-to-vigorous physical activity MVPA and SLP are significantly associated with lower odds of myopia (OR = 0.86, 95% CI: 0.78–0.95, *P* < 0.01; OR = 0.89, 95% CI: 0.81–0.97, *P* < 0.05, respectively), whereas SB and LPA are associated with higher odds of myopia (OR = 1.11, 95% CI: 1.03–1.21, *P* < 0.01; OR = 1.09, 95% CI: 1.02–1.18, *P* < 0.05, respectively). These results are consistent with the LASSO regression findings, suggesting that higher MVPA and sufficient sleep may play protective roles against myopia.

### 3.4 LASSO regression analysis of the association between uncorrected visual acuity and 24-h activity behaviors among middle school students

Seven variables are significantly associated with impaired UCVA in the univariate analysis. Students with parental myopia (χ^2^ = 10.62, *P* < 0.01), higher academic stress (*t* = 6.12, *P* < 0.05), and lower levels of daily outdoor activity (χ^2^ = 6.87, *P* < 0.01) are more likely to exhibit impaired UCVA. In terms of 24-h activity behaviors, MVPA (*t* = 8.29, *P* < 0.01) and shorter SLP (*t* = 8.47, *P* < 0.01) are protective, whereas greater SB (*t* = −8.04, *P* < 0.01) and LPA (*t* = −5.35, *P* < 0.05) are risk factors for impaired vision.

A LASSO (Least Absolute Shrinkage and Selection Operator) regression analysis is conducted to examine the relationship between middle school students‘ uncorrected visual acuity and their 24-h activity behaviors, while controlling for potential covariates such as gender (Male = 1, Female = 0), place of residence (Urban = 1, Rural = 0), parental education level (high school and above = 1, below high school = 0), monthly household income (≥8,000 = 1, < 8,000 = 0), academic stress (mild = 1, moderate = 2, relatively high = 3, severe = 4), parental myopia status (one parent or both parents myopic = 1, neither parent myopic = 0), and outdoor activity time. Prior to the regression analysis, the normality of the 24-h activity behavior variables is assessed using Q-Q plots. Although MVPA (moderate-to-vigorous physical activity) and SB (sedentary behavior) displayed slight right-skewed tendencies, the distributions of MVPA, LPA (light physical activity), SB, and SLP (sleep duration) are generally consistent with normality. Moreover, LASSO regression does not impose strict assumptions regarding data normality, thus the analysis proceeded. The LASSO regression identified: core variables (LPA, MVPA, SB, SLP), potential covariates (parental myopia status, outdoor activity time, and academic stress level) as non-zero predictors, suggesting these behaviors exert differential effects on uncorrected visual acuity. The optimal value of the regularization parameter λ is selected based on the minimum mean squared error from cross-validation (λ = 0.023). The final model revealed that MVPA (β = 0.162), SLP (β = 0.131) and outdoor activity time (β = 0.166) are positively associated with uncorrected visual acuity, whereas SB (β = −0.106), LPA (β = −0.078), parental myopia status β = −0.315), academic stress level (β = −0.113) are negatively associated. Accordingly, the resulting predictive model for middle school students' uncorrected visual acuity is as follows: Uncorrected Visual Acuity = 41.92 – (0.315 × Parental Myopia Status) + (0.166 × Outdoor Activity Time) – (0.113 × Academic Stress Level) + (0.160 × MVPA) + (0.130 × SLP) – (0.105 × SB) – (0.077 × LPA) (Figure 1 shows only core variables).

**Figure 1:**
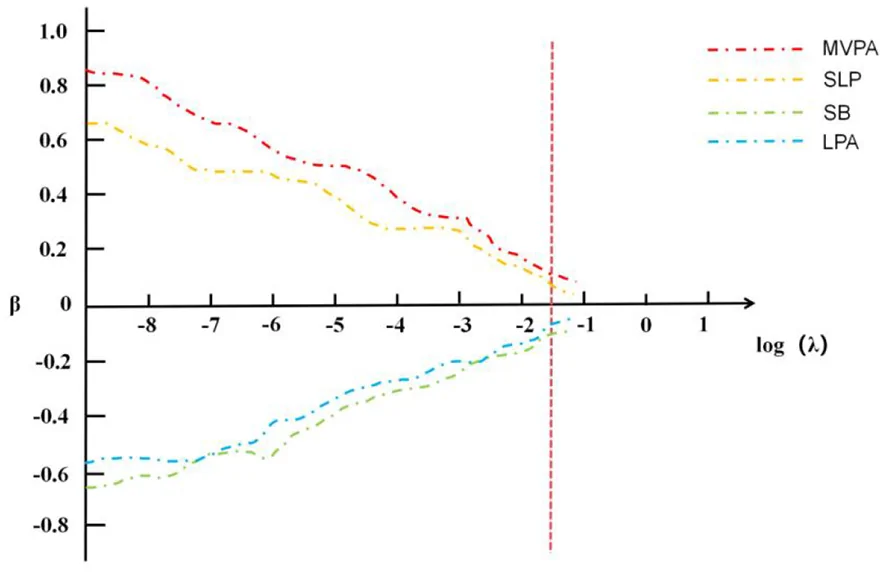
LASSO regression coefficient path map of activity behavior and UCVA in 24 h.

## 4 Discussion

This study explored the relationship between uncorrected visual acuity and 24-h activity behaviors among junior high school students in Tianjin, based on ophthalmic examinations and continuous activity monitoring. Collectively, the present study extends existing myopia-related research through several methodological advances. First, unlike most previous studies relying on self-reported questionnaires or single-behavior indicators, we employed 7-day continuous accelerometer monitoring to objectively capture the full spectrum of 24-h activity behaviors, substantially reducing recall bias and exposure misclassification. Second, by adopting a 24-h movement behavior framework, this study explicitly accounts for the time-constrained and interdependent nature of daily behaviors, providing a more ecologically valid perspective on the behavioral correlates of uncorrected visual acuity. Third, the application of LASSO regression allowed robust variable selection under conditions of multicollinearity among time-based behaviors, offering methodological advantages over traditional regression approaches commonly used in previous studies. Finally, cross-validation of accelerometer-derived measures against questionnaire data using intraclass correlation coefficients further strengthens confidence in the validity of the activity behavior assessment.

Although the direction of associations observed in this study is largely consistent with previous findings, the present results provide important confirmatory evidence based on objective and continuous 24-h activity measurements. Such validation is particularly meaningful given the substantial measurement error and recall bias inherent in questionnaire-based studies. According to the results, the prevalence of myopia among the 1,212 participating students is 73.3%, which is slightly higher than the national average reported in the 2022 survey on myopia among Chinese children and adolescents (71.1%) (10). The detection rate of myopia is lower in boys (71.4%) than in girls (75.6%), a pattern consistent with a trend analysis conducted from 2010 to 2019 among Han Chinese children and adolescents aged 7–18 years, which also reported a higher prevalence of myopia in girls (14). Furthermore, myopia prevalence increased with grade level, and the proportion of students with mild, moderate, and severe visual impairment also rose accordingly, reaching 8.2%, 30.8%, and 39.2%, respectively, by the third year of junior high school. This trend may be attributed to greater physical activity and outdoor time among boys, as they tend to be more active than girls during adolescence. Meanwhile, increasing academic pressure with higher grade levels likely contributes to prolonged near work and reduced outdoor activity time, thereby increasing the risk of myopia.

The World Health Organization (WHO) recommends that children and adolescents minimize sedentary behavior, particularly screen time. According to the most recent Chinese physical activity guidelines for the population released in 2021 (15), children and adolescents aged 6–17 years should avoid prolonged sedentary periods (no more than 1 h at a time), limit daily screen time to no more than 2 h, and engage in at least 1 h of moderate-to-vigorous physical activity (MVPA), preferably outdoors. However, the findings of this study showed that the average durations for LPA (light physical activity), MVPA, SB (sedentary behavior), and SLP (sleep duration) among the 1,212 Tianjin students are 376.09 ± 17.16 min, 38.74 ± 15.36 min, 572.08 ± 51.16 min, and 453.10 ± 47.18 min, respectively—clearly falling short of the recommended guidelines. Similar trends have been reported elsewhere in China. For instance, surveillance data from Anhui Province indicated that over 40% of students engaged in < 1 h of outdoor activity per day (16). In Shanghai, only 5.7% of surveyed children and adolescents met WHO-recommended physical activity levels (17). Furthermore, the *China National Children's Development Status Report 1999–2010* (18) indicated that primary and secondary students slept for an average of 7.61 ± 2.50 h on school days and 7.81 ± 2.56 h on rest days, suggesting that the students in this study may also be experiencing sleep deprivation.

Given that LPA, MVPA, SB, and SLP are time-based variables that are inherently interdependent, traditional regression models may suffer from multicollinearity. To address this, we employed LASSO regression to explore the association between 24-h activity behaviors and uncorrected visual acuity. The results indicated that MVPA and SLP are positively associated with uncorrected visual acuity, while LPA and SB are negatively associated, suggesting that increasing MVPA and sleep duration while reducing sedentary time and LPA may help mitigate the risk of myopia among adolescents. From a 24-h movement behavior perspective, these findings suggest that visual health should not be attributed to any single behavior in isolation, but rather understood as the cumulative and interdependent effects of daily time-use patterns.

In addition to behavior-specific interpretations, consideration of the broader national context may further aid in understanding these findings. China has one of the highest prevalences of myopia among children and adolescents worldwide, with early onset and rapid progression (2, 19). This pattern is generally attributed to a combination of environmental, educational, and institutional factors rather than individual behaviors alone. In China, adolescents are commonly exposed to high academic demands, prolonged near-work activities, and relatively limited outdoor exposure, all of which have been consistently associated with myopia risk in East Asian populations (20, 21). Although nationwide strategies for myopia prevention and control have been implemented in recent years, their effectiveness may be influenced by structural characteristics of the education system and variability in implementation (22). Within this context, the present study provides context-specific evidence by elucidating associations between objectively measured 24-h activity behaviors and uncorrected visual acuity among Chinese adolescents.

Specifically, numerous studies have shown that sleep durations of at least 8 h are protective against myopia. This may be because inadequate sleep disrupts circadian rhythms, interferes with normal axial eye development and ocular tissue repair, and may also be related to hormonal imbalances that impair eye health (23). In addition, cohort studies have demonstrated that physical activity—especially of moderate-to-vigorous intensity—is inversely associated with myopia incidence (22), while sedentary behavior is positively associated with it. Although the underlying physiological mechanisms remain unclear, potential explanations include increased choroidal blood flow due to physical activity, which may inhibit axial elongation of the eye (24). Outdoor activity also helps reduce hyperopic defocus on the peripheral retina, stabilizes retinal image signals, and slows axial elongation—factors that are believed to delay the progression of myopia (25, 26). Conversely, prolonged near work and excessive screen time are strongly associated with increased myopia risk. Supporting this, an isotemporal substitution analysis by Xu Xing and colleagues (27) found that replacing 1 h of sedentary behavior with either 1 h of outdoor physical activity or sleep significantly reduced the risk of visual impairment in primary and secondary school students. These results further underscore the importance of considering daily behaviors within a unified 24-h framework, in which changes in one behavior necessarily occur at the expense of another.

Although significant associations between 24-h activity behaviors and uncorrected visual acuity were observed, the cross-sectional design precludes causal inference, and the findings should be interpreted as correlational. Nevertheless, several plausible mediating and moderating mechanisms may help explain the observed relationships with myopia. Outdoor exposure may mediate the protective association between moderate-to-vigorous physical activity (MVPA) and visual health, as higher ambient light intensity stimulates retinal dopamine release and inhibits excessive axial elongation (28, 29). Thus, the association between MVPA and better visual acuity may reflect increased outdoor light exposure rather than physical activity alone. Conversely, sedentary behavior and light-intensity physical activity may reflect prolonged near-work activities, which increase accommodative demand and retinal hyperopic defocus, thereby promoting axial elongation and myopia development (30, 31). Sleep duration represents another potential mediating pathway, as insufficient sleep has been associated with disrupted circadian regulation and ocular growth, increasing myopia risk (32). In addition, contextual factors such as academic stress and parental myopia may moderate these associations, potentially attenuating the protective effects of physical activity or exacerbating the adverse impact of sedentary behavior (33).

By considering the interdependencies among 24-h activity behaviors, this study provides an integrated analysis of their associations with uncorrected visual acuity, offering both theoretical insights and practical guidance for health promotion among children and adolescents. And from a practical perspective, the use of objective 24-h activity monitoring may facilitate more precise identification of at-risk behavioral patterns related to visual health. This approach provides a methodological foundation for future intervention studies aiming to optimize daily time allocation rather than targeting single behaviors alone. However, several limitations should be noted:

1. The study sample is limited to junior high school students in Tianjin, which may restrict the generalizability of the findings; future research should expand the sample size and include students from other regions.
2. In this study, SLP is calculated as 1,440 mins minus total PA and SB duration. Using accelerometer non-wear time as a proxy for sleep, this method may overestimate sleep by including non-sleep activities (e.g., bathing, dressing). Future research should use precise tools (wrist-worn monitors, small-sample polysomnography) to reduce bias, and analyze sleep pattern differences across myopia severity to clarify sleep's protective role in visual health.
3. Due to the cross-sectional design, causal relationships between 24-h activity behaviors and myopia cannot be established. Future longitudinal and interventional studies are required to clarify causal pathways and potential mediating and moderating mechanisms.
4. Research on the relationship between light physical activity and myopia remains limited, and future studies should aim to explore this area in more depth.
5. Future studies should consider integrating questionnaire design with vision stratification to allow for more refined subgroup comparisons.

## Data Availability

The datasets presented in this article are not readily available because National-level confidential database. Requests to access the datasets should be directed to Zhao Yanan 1154317556@qq.com.
